# 4-Bromo-3-methyl­anilinium hydrogen sulfate

**DOI:** 10.1107/S160053680903493X

**Published:** 2009-09-09

**Authors:** Li Zhang

**Affiliations:** a Ordered Matter Science Research Center, College of Chemistry and Chemical, Engineering, Southeast University, Nanjing 210096, People’s Republic of China

## Abstract

In the cation of the title compound, C_7_H_9_BrN^+^·HSO_4_^−^, the amino N atom is protonated. In the crystal, inter­molecular O—H⋯O and N—H⋯O hydrogen bonds generate an infinite two-dimensional network parallel to (001).

## Related literature

For the structures of amino derivatives, see: Fu *et al.* (2007 ▶, 2008 ▶); Fu & Xiong (2008 ▶). Amino derivatives are used in the construction of metal-organic frameworks. For applications of metal-organic coordination compounds, see: Chen *et al.* (2001 ▶); Xiong *et al.* (1999 ▶); Xie *et al.* (2002 ▶); Zhao *et al.* (2004 ▶); Wang *et al.* (2002 ▶). 
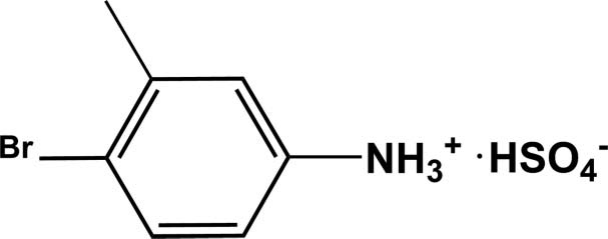


## Experimental

### 

#### Crystal data

C_7_H_9_BrN^+^·HSO_4_^−^
*M* *_r_* = 284.13
Triclinic, 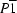

*a* = 4.9448 (10) Å
*b* = 6.4084 (13) Å
*c* = 16.674 (3) Å
α = 98.92 (3)°
β = 96.22 (3)°
γ = 100.01 (3)°
*V* = 509.04 (17) Å^3^
*Z* = 2
Mo *K*α radiation
μ = 4.23 mm^−1^
*T* = 298 K
0.40 × 0.05 × 0.05 mm

#### Data collection

Rigaku Mercury2 diffractometer
Absorption correction: multi-scan (*CrystalClear*; Rigaku, 2005 ▶) *T*_min_ = 0.910, *T*_max_ = 1.000
5279 measured reflections
2323 independent reflections
1804 reflections with *I* > 2σ(*I*)
*R*_int_ = 0.053

#### Refinement

*R*[*F* ^2^ > 2σ(*F* ^2^)] = 0.049
*wR*(*F* ^2^) = 0.115
*S* = 1.07
2323 reflections
129 parameters
H-atom parameters constrained
Δρ_max_ = 0.40 e Å^−3^
Δρ_min_ = −0.67 e Å^−3^

### 

Data collection: *CrystalClear* (Rigaku, 2005 ▶); cell refinement: *CrystalClear*; data reduction: *CrystalClear*; program(s) used to solve structure: *SHELXS97* (Sheldrick, 2008 ▶); program(s) used to refine structure: *SHELXL97* (Sheldrick, 2008 ▶); molecular graphics: *SHELXTL* (Sheldrick, 2008 ▶); software used to prepare material for publication: *SHELXTL*.

## Supplementary Material

Crystal structure: contains datablocks I, global. DOI: 10.1107/S160053680903493X/pv2203sup1.cif

Structure factors: contains datablocks I. DOI: 10.1107/S160053680903493X/pv2203Isup2.hkl

Additional supplementary materials:  crystallographic information; 3D view; checkCIF report

## Figures and Tables

**Table 1:** Hydrogen-bond geometry (Å, °)

*D*—H⋯*A*	*D*—H	H⋯*A*	*D*⋯*A*	*D*—H⋯*A*
N1—H1*A*⋯O3^i^	0.89	1.90	2.767 (3)	166
N1—H1*B*⋯O2^ii^	0.89	1.91	2.797 (4)	173
O1—H1⋯O4^iii^	0.82	1.84	2.650 (3)	168
N1—H1*C*⋯O4	0.89	2.09	2.829 (4)	140

Symmetry codes: (i) 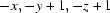; (ii) 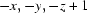; (iii) 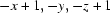.

**Fig. 1.**
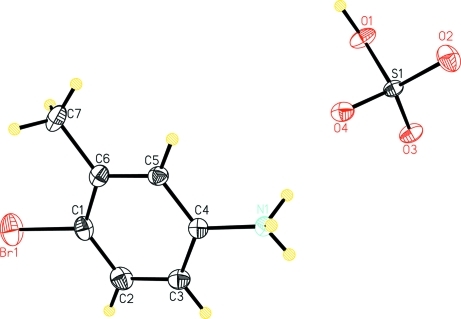
A view of the title compound with the atomic numbering scheme. Displacement ellipsoids have been drawn at the 30% probability level.

**Fig. 2.**
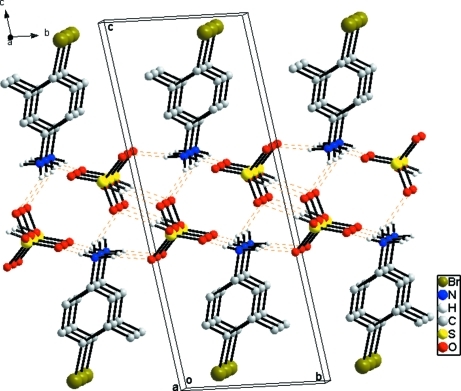
The crystal packing of the title compound, viewed along the a axis showing hydrogen bonding (dotted line); H atoms not involved in hydrogen bonding have been omitted for clarity.

## supplementary crystallographic information

### Comment

The construction of metal-organic coordination compounds has attracted much attention owing to potential functions, such as permittivity, fluorescence, magnetism and optical properties (Wang *et al.* 2002; Fu *et al.*, 2008; Chen *et al.*, 2001; Xie *et al.*, 2002; Zhao *et al.*,2004; Xiong *et al.*, 1999). Amino derivatives are a class of excellent ligands for the construction of novel metal-organic frameworks. (Fu *et al.*, 2007; Fu & Xiong 2008). We report here the crystal structure of the title compound, 4-bromo-3-methylanilinium bisulfate.

In the title compound (Fig.1), The amino N atoms are protonated. In the crystal structure, all the amine group H atoms and HSO_4_^-^ H atoms are involved in N—H···O and O—H···O hydrogen bonds (Table 1) with O atoms of HSO_4_^-^ anion. These hydrogen bonds link the ionic units into a two-dimensional network (Fig. 2).

### Experimental

The commercial 4-bromo-3-methylaniline (3 mmol) and H_2_SO_4_ (0.5 ml) were dissolved in ethanol (20 ml). Colourless block-shaped crystals of the title compound suitable for X-ray analysis were obtained by slow evaporation at room temperature.

### Refinement

All H atoms attached to C, O and N atoms were fixed geometrically and treated as riding with C–H = 0.93 Å (aromatic), C–H = 0.96 Å (methyl), O–H = 0.82 Å and N–H = 0.89 Å with *U*_iso_(H) =1.2*U*_eq_(C) and *U*_iso_(H) =1.5*U*_eq_(O or N).

### Crystal data

**Table:**

C_7_H_9_BrN^+^·HSO_4_^−^	*Z* = 2
*M**_r_* = 284.13	*F*(000) = 284
Triclinic, *P*1	*D*_x_ = 1.854 Mg m^−^^3^
Hall symbol: -P 1	Mo *K*α radiation, λ = 0.71073 Å
*a* = 4.9448 (10) Å	Cell parameters from 1804 reflections
*b* = 6.4084 (13) Å	θ = 3.3–27.5°
*c* = 16.674 (3) Å	µ = 4.23 mm^−^^1^
α = 98.92 (3)°	*T* = 298 K
β = 96.22 (3)°	Block, colorless
γ = 100.01 (3)°	0.40 × 0.05 × 0.05 mm
*V* = 509.04 (17) Å^3^	

### Data collection

**Table:**

Rigaku Mercury2 diffractometer	2323 independent reflections
Radiation source: fine-focus sealed tube	1804 reflections with *I* > 2σ(*I*)
graphite	*R*_int_ = 0.053
Detector resolution: 13.6612 pixels mm^-1^	θ_max_ = 27.5°, θ_min_ = 3.3°
CCD profile fitting scans	*h* = −6→6
Absorption correction: multi-scan (*CrystalClear*; Rigaku, 2005)	*k* = −8→8
*T*_min_ = 0.910, *T*_max_ = 1.000	*l* = −21→21
5279 measured reflections	

### Refinement

**Table:**

Refinement on *F*^2^	Primary atom site location: structure-invariant direct methods
Least-squares matrix: full	Secondary atom site location: difference Fourier map
*R*[*F*^2^ > 2σ(*F*^2^)] = 0.049	Hydrogen site location: inferred from neighbouring sites
*wR*(*F*^2^) = 0.115	H-atom parameters constrained
*S* = 1.07	*w* = 1/[σ^2^(*F*_o_^2^) + (0.0426*P*)^2^ + 0.1536*P*] where *P* = (*F*_o_^2^ + 2*F*_c_^2^)/3
2323 reflections	(Δ/σ)_max_ < 0.001
129 parameters	Δρ_max_ = 0.40 e Å^−^^3^
0 restraints	Δρ_min_ = −0.67 e Å^−^^3^

### Special details

**Table:**

Geometry. All e.s.d.'s (except the e.s.d. in the dihedral angle between two l.s. planes) are estimated using the full covariance matrix. The cell e.s.d.'s are taken into account individually in the estimation of e.s.d.'s in distances, angles and torsion angles; correlations between e.s.d.'s in cell parameters are only used when they are defined by crystal symmetry. An approximate (isotropic) treatment of cell e.s.d.'s is used for estimating e.s.d.'s involving l.s. planes.
Refinement. Refinement of *F*^2^ against ALL reflections. The weighted *R*-factor *wR* and goodness of fit *S* are based on *F*^2^, conventional *R*-factors *R* are based on *F*, with *F* set to zero for negative *F*^2^. The threshold expression of *F*^2^ > σ(*F*^2^) is used only for calculating *R*-factors(gt) *etc*. and is not relevant to the choice of reflections for refinement. *R*-factors based on *F*^2^ are statistically about twice as large as those based on *F*, and *R*- factors based on ALL data will be even larger.

### Fractional atomic coordinates and isotropic or equivalent isotropic displacement parameters (Å^2^)

**Table:**

	*x*	*y*	*z*	*U*_iso_*/*U*_eq_	
Br1	0.74330 (10)	0.74731 (8)	0.96412 (3)	0.0635 (2)	
N1	0.0682 (6)	0.3866 (4)	0.63055 (17)	0.0278 (6)	
H1A	−0.0452	0.4741	0.6182	0.042*	
H1B	−0.0308	0.2561	0.6299	0.042*	
H1C	0.1857	0.3782	0.5939	0.042*	
C1	0.5271 (7)	0.6260 (6)	0.8613 (2)	0.0355 (8)	
C4	0.2233 (7)	0.4704 (5)	0.7120 (2)	0.0273 (7)	
C5	0.4016 (7)	0.3520 (5)	0.7448 (2)	0.0290 (7)	
H5	0.4154	0.2193	0.7156	0.035*	
C3	0.1915 (7)	0.6643 (5)	0.7535 (2)	0.0329 (8)	
H3	0.0696	0.7418	0.7309	0.039*	
C6	0.5593 (7)	0.4282 (6)	0.8206 (2)	0.0309 (8)	
C2	0.3444 (8)	0.7420 (6)	0.8295 (2)	0.0404 (9)	
H2	0.3247	0.8723	0.8594	0.049*	
C7	0.7547 (8)	0.2971 (7)	0.8549 (3)	0.0458 (10)	
H7A	0.9411	0.3782	0.8635	0.069*	
H7B	0.7431	0.1659	0.8169	0.069*	
H7C	0.7043	0.2638	0.9061	0.069*	
S1	0.37322 (15)	0.18764 (12)	0.42345 (5)	0.0231 (2)	
O1	0.6927 (5)	0.1969 (4)	0.43590 (16)	0.0379 (6)	
H1	0.7257	0.0932	0.4558	0.057*	
O2	0.2512 (6)	0.0108 (4)	0.35840 (16)	0.0433 (7)	
O3	0.3537 (5)	0.3945 (3)	0.40391 (16)	0.0328 (6)	
O4	0.2770 (5)	0.1540 (4)	0.50052 (14)	0.0305 (5)	

### Atomic displacement parameters (Å^2^)

**Table:**

	*U*^11^	*U*^22^	*U*^33^	*U*^12^	*U*^13^	*U*^23^
Br1	0.0640 (3)	0.0704 (4)	0.0396 (3)	−0.0081 (2)	−0.0124 (2)	−0.0034 (2)
N1	0.0312 (15)	0.0272 (15)	0.0268 (14)	0.0091 (11)	0.0038 (11)	0.0062 (12)
C1	0.0342 (19)	0.038 (2)	0.0290 (18)	−0.0039 (15)	0.0024 (15)	0.0041 (16)
C4	0.0278 (17)	0.0235 (17)	0.0296 (18)	0.0015 (13)	0.0040 (13)	0.0058 (14)
C5	0.0307 (17)	0.0267 (17)	0.0319 (18)	0.0076 (14)	0.0071 (14)	0.0080 (15)
C3	0.0350 (19)	0.0261 (18)	0.038 (2)	0.0064 (14)	0.0067 (15)	0.0066 (16)
C6	0.0254 (17)	0.0352 (19)	0.0342 (19)	0.0017 (14)	0.0067 (14)	0.0156 (16)
C2	0.050 (2)	0.0265 (19)	0.041 (2)	0.0040 (16)	0.0063 (18)	−0.0014 (17)
C7	0.044 (2)	0.055 (3)	0.043 (2)	0.0132 (18)	−0.0031 (18)	0.023 (2)
S1	0.0207 (4)	0.0192 (4)	0.0313 (4)	0.0042 (3)	0.0043 (3)	0.0091 (3)
O1	0.0225 (12)	0.0427 (15)	0.0590 (17)	0.0108 (10)	0.0136 (11)	0.0304 (13)
O2	0.0570 (17)	0.0272 (13)	0.0392 (15)	−0.0020 (11)	0.0044 (13)	−0.0009 (12)
O3	0.0303 (13)	0.0235 (12)	0.0486 (15)	0.0056 (10)	0.0055 (11)	0.0181 (11)
O4	0.0318 (13)	0.0285 (13)	0.0369 (14)	0.0092 (10)	0.0122 (10)	0.0145 (11)

### Geometric parameters (Å, °)

**Table:**

Br1—C1	1.894 (4)	C3—H3	0.9300
N1—C4	1.458 (4)	C6—C7	1.509 (5)
N1—H1A	0.8900	C2—H2	0.9300
N1—H1B	0.8900	C7—H7A	0.9600
N1—H1C	0.8900	C7—H7B	0.9600
C1—C2	1.379 (6)	C7—H7C	0.9600
C1—C6	1.386 (5)	S1—O3	1.430 (2)
C4—C3	1.369 (5)	S1—O2	1.437 (3)
C4—C5	1.382 (5)	S1—O4	1.452 (2)
C5—C6	1.380 (5)	S1—O1	1.560 (2)
C5—H5	0.9300	O1—H1	0.8200
C3—C2	1.376 (5)		
			
C4—N1—H1A	109.5	C5—C6—C7	119.9 (3)
C4—N1—H1B	109.5	C1—C6—C7	123.4 (3)
H1A—N1—H1B	109.5	C3—C2—C1	119.8 (3)
C4—N1—H1C	109.5	C3—C2—H2	120.1
H1A—N1—H1C	109.5	C1—C2—H2	120.1
H1B—N1—H1C	109.5	C6—C7—H7A	109.5
C2—C1—C6	122.6 (3)	C6—C7—H7B	109.5
C2—C1—Br1	117.8 (3)	H7A—C7—H7B	109.5
C6—C1—Br1	119.6 (3)	C6—C7—H7C	109.5
C3—C4—C5	121.7 (3)	H7A—C7—H7C	109.5
C3—C4—N1	119.5 (3)	H7B—C7—H7C	109.5
C5—C4—N1	118.8 (3)	O3—S1—O2	114.06 (16)
C6—C5—C4	120.8 (3)	O3—S1—O4	113.59 (14)
C6—C5—H5	119.6	O2—S1—O4	111.41 (15)
C4—C5—H5	119.6	O3—S1—O1	102.60 (13)
C4—C3—C2	118.4 (3)	O2—S1—O1	107.73 (16)
C4—C3—H3	120.8	O4—S1—O1	106.61 (14)
C2—C3—H3	120.8	S1—O1—H1	109.5
C5—C6—C1	116.7 (3)		

### Hydrogen-bond geometry (Å, °)

**Table:**

*D*—H···*A*	*D*—H	H···*A*	*D*···*A*	*D*—H···*A*
N1—H1A···O3^i^	0.89	1.90	2.767 (3)	166
N1—H1B···O2^ii^	0.89	1.91	2.797 (4)	173
O1—H1···O4^iii^	0.82	1.84	2.650 (3)	168
N1—H1C···O4	0.89	2.09	2.829 (4)	140

Symmetry codes: (i) −*x*, −*y*+1, −*z*+1; (ii) −*x*, −*y*, −*z*+1; (iii) −*x*+1, −*y*, −*z*+1.
